# Semi-Quantitative Mass Spectrometry in AML Cells Identifies New Non-Genomic Targets of the EZH2 Methyltransferase

**DOI:** 10.3390/ijms18071440

**Published:** 2017-07-05

**Authors:** Yordan Sbirkov, Colin Kwok, Amandeep Bhamra, Andrew J. Thompson, Veronica Gil, Arthur Zelent, Kevin Petrie

**Affiliations:** 1 Theodor Boveri Institute and Comprehensive Cancer Center Mainfranken, Biocenter, University of Würzburg, Würzburg 97074, Germany; yordan.sbirkov@uni-wuerzburg.de; 2 Division of Clinical Studies, Institute of Cancer Research, London SW7 3RP, UK; colin.kwok@icr.ac.uk (C.K.); veronica.gil@icr.ac.uk (V.G.); 3 UCL Cancer Institute, Paul O’Gormon Building, London WC1E 6DD, UK; a.bhamra@ucl.ac.uk; 4 Proteomics and Metabolomics Core Facility, Institute of Cancer Research, London SW7 3RP, UK; a.thompson@invivaconsulting.com; 5 Miller School of Medicine, University of Miami, Miami, FL 33136, USA; a.zelent@med.miami.edu; 6 Faculty of Natural Sciences, University of Stirling, Stirling FK9 4LA, UK

**Keywords:** acute myeloid leukaemia, EZH2, mass spectrometry, methylation, eEF1A1

## Abstract

Alterations to the gene encoding the EZH2 (KMT6A) methyltransferase, including both gain-of-function and loss-of-function, have been linked to a variety of haematological malignancies and solid tumours, suggesting a complex, context-dependent role of this methyltransferase. The successful implementation of molecularly targeted therapies against EZH2 requires a greater understanding of the potential mechanisms by which EZH2 contributes to cancer. One aspect of this effort is the mapping of EZH2 partner proteins and cellular targets. To this end we performed affinity-purification mass spectrometry in the FAB-M2 HL-60 acute myeloid leukaemia (AML) cell line before and after all-*trans* retinoic acid-induced differentiation. These studies identified new EZH2 interaction partners and potential non-histone substrates for EZH2-mediated methylation. Our results suggest that EZH2 is involved in the regulation of translation through interactions with a number of RNA binding proteins and by methylating key components of protein synthesis such as eEF1A1. Given that deregulated mRNA translation is a frequent feature of cancer and that eEF1A1 is highly expressed in many human tumours, these findings present new possibilities for the therapeutic targeting of EZH2 in AML.

## 1. Introduction

Acute myeloid leukaemia (AML) is the most commonly occurring acute haematological malignancy in adults, representing more than 80% of all leukaemias in patients over 60 years of age [1]. AML also accounts for 15–20% of childhood leukaemia cases, making it the second most common acute leukaemia in children [2,3,4]. Despite advances in diagnosis, stratification and treatment, the disease remains largely incurable (in 60–65% of patients <60 years and 85–95% of patients >60 years [5]), and overall 5-year survival rates remain poor at only 25% [4,6]. Furthermore, treatment outcomes for relapsed patients are low, with complete remission rates under 25% [7,8]. New treatment options are therefore urgently required.

Epigenetic events play a central role in normal development and differentiation, and it is unsurprising that mutation and/or deregulation of DNA and histone modifiers is a frequent event in cancer [9]. Epigenetic enzymes that have been implicated in the promotion of haematological malignancies include *MLL*, translocations of which occur in approximately 80% of infant leukaemias and 5–10% of adult AML [10], resulting in gain-of-function mutations of the encoded H3K4 methyltransferase. Other recently described examples include mutation of DNMT3A (which occurs in approximately 20% of AML patients) [11], aberrant expression of the LSD1 (KDM1A) demethylase (which is strongly implicated in AML) [12,13,14], and overexpression of histone deacetylase 9 (HDAC9), which is associated with leukaemia and lymphoma [15,16]. Perhaps the most widely-studied epigenetic modifiers, however, are the Polycomb group of proteins (PcG), which form two distinct multiprotein repressive complexes, PRC1 and PRC2. PRC1 is required for the ubiquitination of histone H2A lysine 119, which proceeds via Ring1a or Ring1b E3 ligases. The core PRC1 subunit BMI1 (also known as PCGF4 or RNF51) is required for cancer stem cell maintenance [17] and its overexpression has been implicated in leukaemia and lymphoma. The canonical function of PRC2 is to catalyse the methylation of histone H3 lysine 27 (H3K27) and contains either the EZH1 or EZH2 methyltransferases. EZH2 has been the subject of intense research in recent years due to its role in a wide range of cancers and to its potential as a therapeutic target [18].

In addition to being overexpressed in a number of solid tumours, EZH2 is also frequently mutated in haematological malignancies. In contrast to the selection for activating point mutations (Y641 and A677) in the SET (Suppressor of variegation, Enhancer of Zeste, Trithorax) domain of EZH2 described in B-cell malignancies [19,20,21], a range of loss-of-function aberrations (including in other PRC2 members) have been found in 25% of T-cell acute lymphoblastic leukaemia (ALL) cases, 3% of primary AML, 29% of secondary AML, and 15% of myeloproliferative disorders [22,23,24]. Importantly, all these perturbations have been shown to lead to poor prognosis and diminished overall survival [23,25,26]. In addition to mutation of EZH2 in AML, evidence strongly suggests its aberrant expression can promote self-renewal of leukaemic stem cells and block differentiation [27,28,29]. Deregulation of EZH2/PRC2 function can therefore occur in a number of ways, pointing to a complex role that may be dependent on the cell type and on the stage of hematopoietic development at which mutations or deregulation of expression occur.

In order to improve both our understanding of the dynamic functions of EZH2 as well as its potential as a therapeutic target, it is important to identify proteins that interact with the core PRC2 complex, as well as non-histone enzymatic substrates. Here, it is important to note that none of the four core PRC2 components (EZH2, SUZ12, EED, and AEBP1/2) has a DNA binding domain and that the PRC2 complex depends on factors such as JARID2, YY1, or AEBP2 [30], and/or non-coding RNAs such as *XIST* and *HOTAIR* to be recruited to specific loci [31]. Non-canonical EZH2-containing PRC2 activities identified thus far have included scaffold functions and lysine modifications of non-histone proteins, including EZH2 bridging of β-catenin and TCF7 (formerly named Tcf1, T cell factor 1) [32], interaction with RelA and RelB [33], methylation of androgen receptor [34], as well as interactions with STAT3 [35,36], GATA4 [37], RORα [38], and Talin1 [39]. Therefore, using the well-characterised HL-60 cell line as a model system [40], we performed mass spectrometry (MS) as an unbiased approach to quantitatively investigate potential modulators (recruiters or co-repressors) and enzymatic targets of EZH2 in the context of AML. We additionally investigated whether these interactions were modulated in response to all-*trans* retinoic acid (AtRA)-induced myeloid differentiation.

## 2. Results

**Figure 1:**
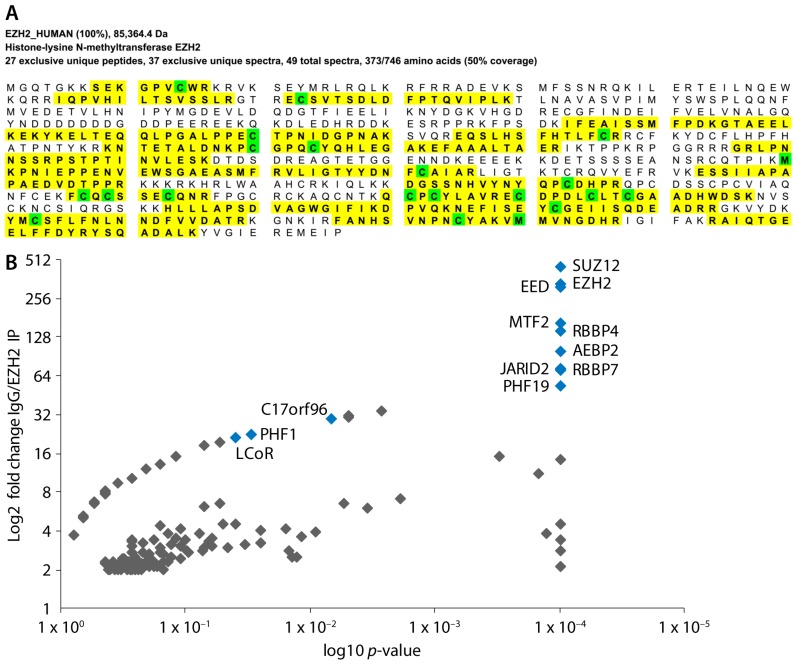
Affinity-purification mass spectrometry coverage of EZH2 and enrichment of PRC2 member proteins. (**A**) Sequence coverage of EZH2 (detected peptides are highlighted in yellow and amino acids with post-translational modifications are shown in green) from a representative mass spectrometry run (peptide threshold 95%); (**B**) Scatter plot of hits from all five mass spectrometry experiments passing a 2-fold change cut-off showing enrichment of core PRC2 components (shown in blue).

We performed five separate MS experiments that comprised a total of five IgG controls and seven EZH2 immunoprecipitations (IP), and one IP with an antibody raised against pan-methyl lysine. Good peptide coverage of EZH2 was achieved in the MS runs (going up to 50% coverage based on peptide assignment of at least 95% peptide threshold using the Peptide Prophet algorithm [41]) and all core components of the PRC2 complex were found to be significantly enriched (highlighted in blue) (Figure 1).

### 2.1. The EZH2 Interactome in HL-60 Cells

**Figure 2:**
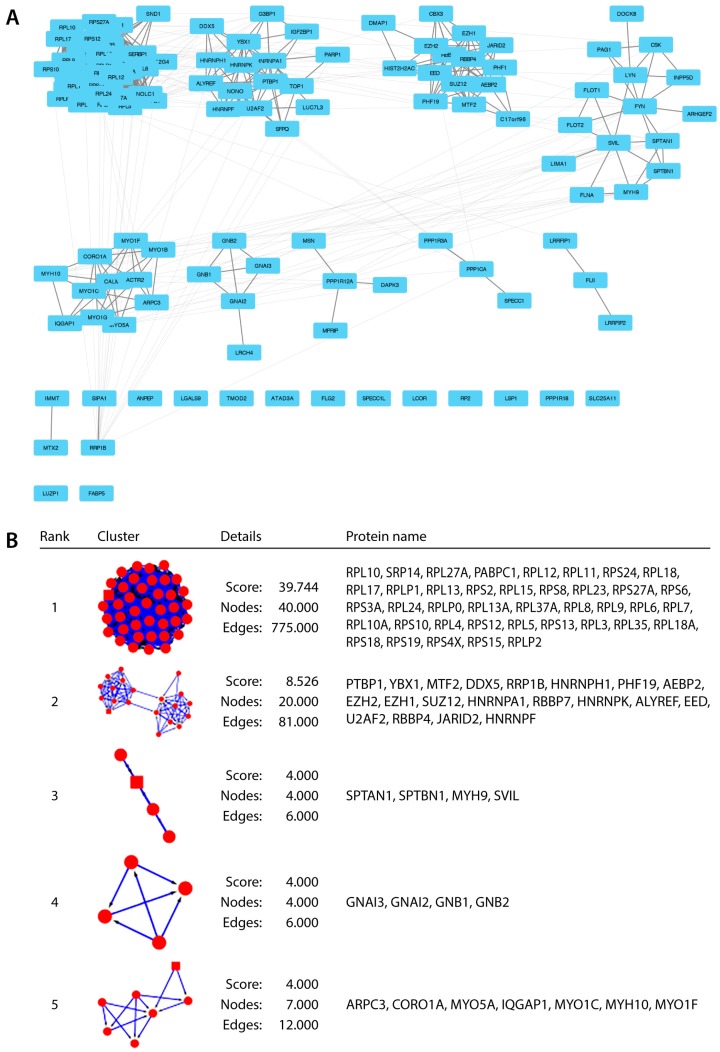
The EZH2 interactome in HL-60 acute myeloid leukaemia (AML) cells. (**A**) Network analysis of EZH2 interactome (2-fold change over IgG and 0.8 SAINTexpress score) showing related groups of proteins as clustered by Cytoscape (Makarov Clustering Algorithm in clusterMaker). Groups of PRC2 interacting proteins (third node from the left), as well as for RNA binding proteins involved in translation and splicing (first and second nodes on the top) can be distinguished; (**B**) MCODE clusters with protein symbols in each node confirming strong enrichment for RNA binding proteins (cluster 1) and known PRC2 associated members (cluster 2).

The first part of this study focussed on building the first EZH2 interactome in AML cells and on quantifying potential changes upon short (overnight) stimulation with AtRA. For this analysis, we considered all seven EZH2 IP and compared the protein abundance to the one found in all five IgG IP (a stringent 2-fold change cut-off was applied in this case) identifying 181 proteins interacting with EZH2. After further filtering (CRAPome [42] and SAINTexpress [43]) and comparison to recently published PcG complexome data [44] (Figure S1), we identified 143 proteins that co-IP with EZH2 in AML cells at high confidence (>0.8 SAINTexpress score). In order to highlight potential molecular complexes among the EZH2 interactors, clustering analysis was then performed with the Cluster maker [45] (MCL cluster), MCODE [46], and ClusterONE [47] plugins in Cytoscape [48], showing one main cluster of proteins and two smaller ones (Figure 2 and Figure S2).

**Figure 3:**
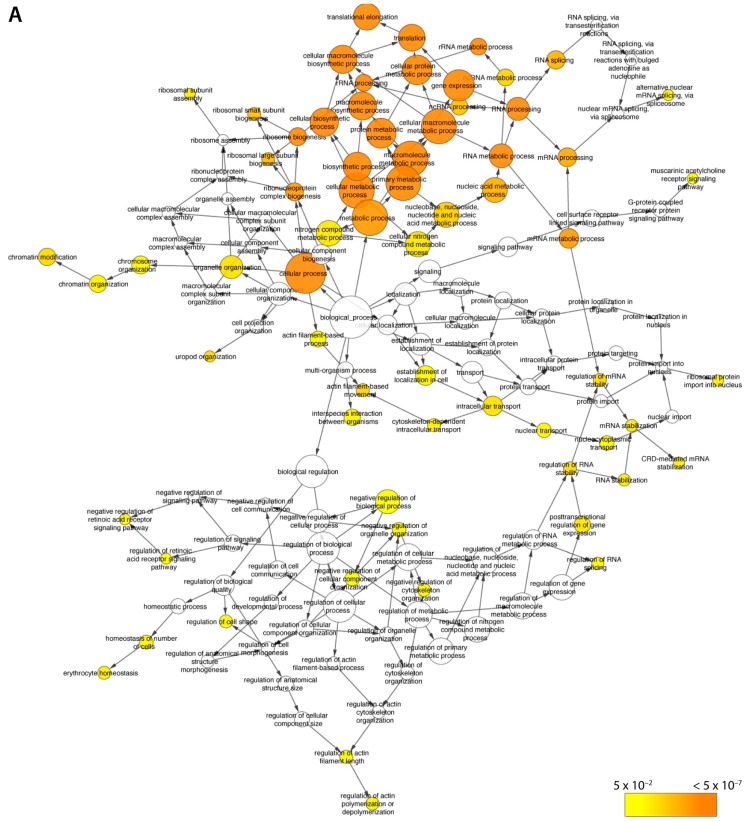

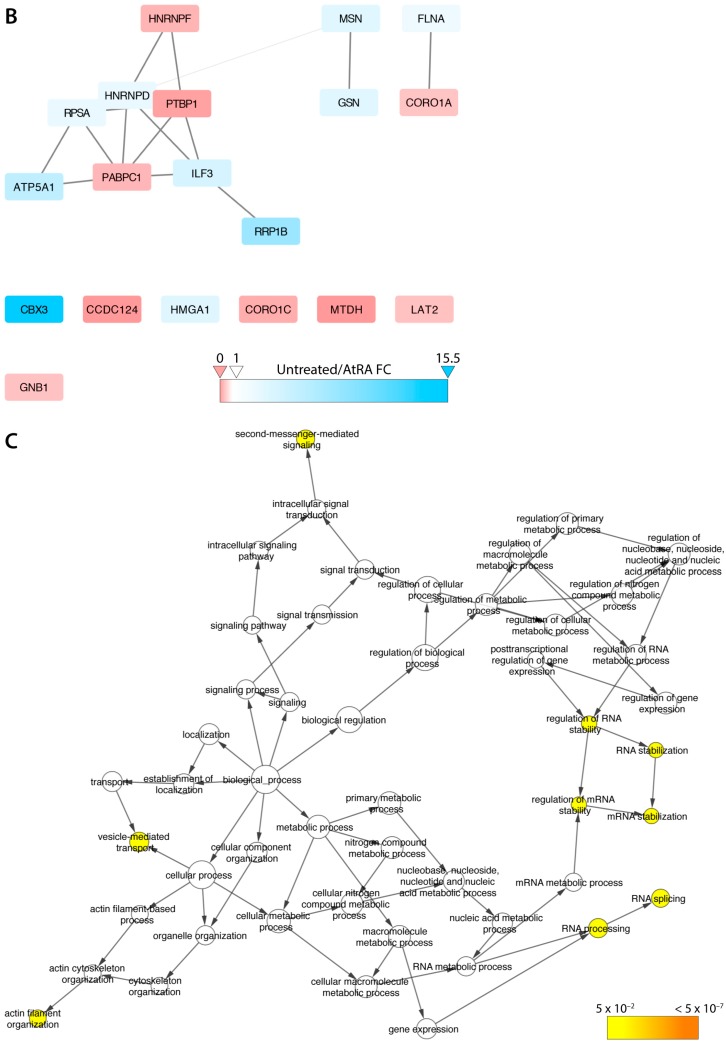
Gene ontology analysis of EZH2 interactome. (**A**) Gene ontology (Cytoscape–BinGO) showing significant enrichment for proteins involved in translation, RNA splicing, and gene expression. Node colour corresponds to *p*-value (see scale). Gene ontology analysis of EZH2 interactome (cont.); (**B**) Quantitative network analysis of EZH2-interacting proteins changing their relative frequency of interaction upon differentiation. Blue-coloured proteins represent ones found to co-IP with EZH2 at higher frequency in untreated HL-60 cells, whereas pink-coloured “preys” were found enriched in cells treated with AtRA; (**C**) Gene ontology (GO) analysis of proteins that change their interaction frequency with EZH2 upon AtRA-driven myeloid differentiation suggesting involvement in RNA metabolism, splicing, and control of gene expression.

Further gene ontology (GO) analysis of hits using BinGO [49], David [50], and STRING [51] revealed a strong enrichment for proteins involved in RNA processing (splicing, mRNA metabolic processes) and translation (ribosomal proteins) located in the biggest cluster and one of the two smaller ones (Figure 3A). These results are in agreement with, and expand upon the most comprehensive proteomics dataset on PcG proteins published to date [44]. Importantly, this analysis also identified a major cluster of interacting proteins that are involved in gene expression and chromatin modifications consisting primarily of PRC2 members (the second small cluster from Figure 2 and Figure S2B,C), as well as several ribosomal proteins. Among those, there were several EZH2-recruiting proteins that have not been previously demonstrated to interact with EZH2 in the context of AML, such as PHF1, PHF19, LCOR, and EPOP (also known as C17orf96). Of note, several other proteins implicated in transcriptional control were also identified including THOC4/ALYREF, DDX5, DMAP1, HNRNPK, NONO, SFPQ, FLII, PARP1, PABP1, U2AFA, PTBP1, and YBX1 (Table 1).

### 2.2. Changes in the EZH2 Interactome in Response to AtRA

We next examined alterations to EZH2 interactions in response to myeloid differentiation. Of the seven EZH2 IP mentioned above, four were of untreated HL-60 cells and three of HL-60 cells stimulated with 0.1 µM AtRA. All runs were again merged and relative quantitation was performed based on exclusive spectrum count. Initially, 82 proteins with a greater than 2-fold difference between the conditions were identified, which were found to be at least 1.5 times more abundant in all 7 EZH2 IP versus all five IgG IP. Nevertheless, after filtering of the hits, 19 proteins were identified as selectively interacting with EZH2 upon AtRA-induced differentiation (Table 2 and Figure 3B). GO and network analyses (Figure 3C) showed strong enrichment for RNA binding proteins (13 out of 19, FDR = 4.86 × 10^−8^), with a number of these hits being involved in mRNA splicing (HNRNPD, HNRNPF, PABPC1, PTBP1, CCDC124) and transcription regulation (CBX3, HMGA, ILF3, MTDH). Of note, none of the genes encoding these proteins were found to display changes in gene expression in accordance with increased or decreased bait-prey interactions following 72 h of treatment with AtRA (unpublished data). Assuming consistent protein stability, this suggests that the changes in the EZH2 interactome identified here are unlikely to result from altered gene expression levels of any of the preys identified.

### 2.3. Identification of Enzymatic Targets of EZH2

**Figure 4:**
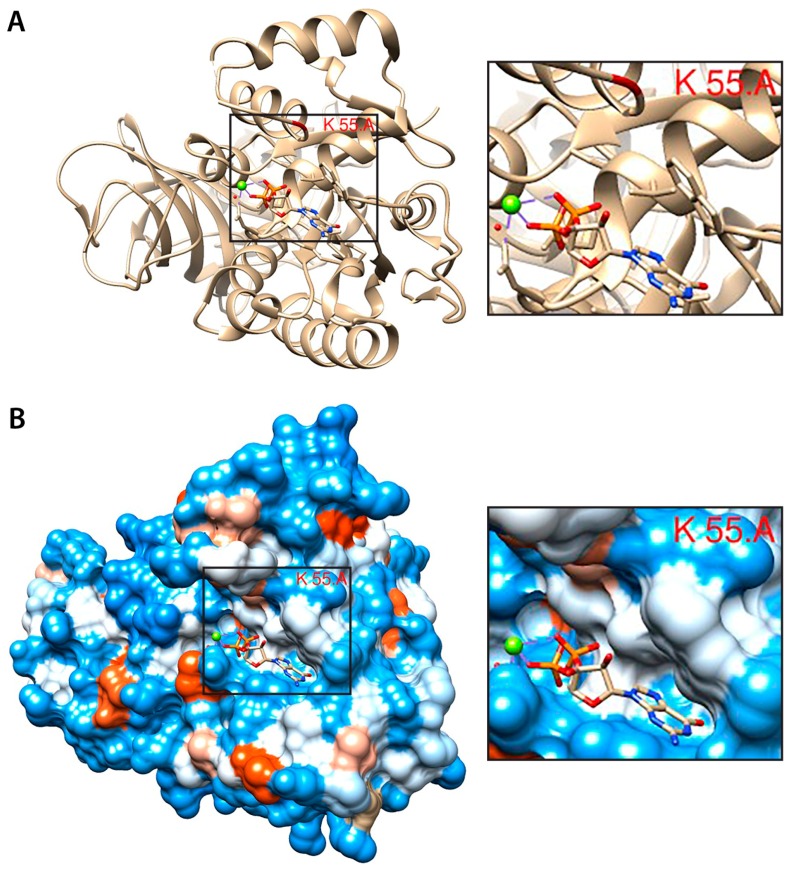
Structure of eEF1A1 and putative EZH2 methylation target site. (**A**) Ribbon structure view of mammalian eEF1A monomer showing close proximity of Lys 55 (highlighted in red) to the GTP/GDP (Guanosine-5′-tri/diphosphate) binding pocket (with a GDP molecule bound in the pocket); (**B**) Hydrophobicity surface view (hydrophobic, red; hydrophilic, blue; neutral, white) of eEF1A1 showing that K55 is in a hydrophilic (blue colour) region close to GTP/GDP binding pocket that is potentially accessible for post-translational modifications and methylation by EZH2.

Finally, we sought to identify potential non-histone targets of EZH2 in AML. Four of the five MS runs (six EZH2 IP and four IgG control IP) were re-run in order to detect methylated peptides. Relatively few proteins modified by mono-, di-, or tri-methylation were detected (32 in total, 24 with modified Lysine residues, eight with Arginine methylation). After filtering for proteins identified in the EZH2 interactome (Figure 2), potential hits for lysine methylation identified with high confidence (99.9% proteins threshold and 80–95% peptide threshold) included EZH2 itself (K735^me1^), SUZ12 (K4^me1^), CBX3 (K142^me1^), Histone H4 (K20^me2^), eEF1A1 (K55^me2^), and ADT2 (also known as SLC25A5) (K51^me3^). (Table 3 and Table 4, Figure 4 and Figure S3).

In order to validate candidate EZH2 targets, another pull-down was performed with a pan-methylated lysine antibody in parallel with the EZH2 IP (data not shown). Although relatively few proteins (25 proteins that had at least 1.5 times more total peptides than in the IgG control) were found to co-IP with this antibody (at high confidence thresholds), this experiment confirmed that eEF1A1 and ADT2 are indeed methylated, suggesting that these two proteins may be direct EZH2 methylation targets (i.e., they co-IP with EZH2 in at least two experiments, they are immunoprecipitated with the pan-methyl lysine antibody, and methylation sites were found by mass spectrometry analysis). Of note, there were six other potential hits from the anti-pan-methylated antibody pull-down that were also found to co-IP with EZH2 (among the final 145 hits)—three other ribosomal proteins (RPL24, RPL35, RPS27A), as well as SPTB2, LUC7L3, and SFPQ (a transcriptional co-repressor). Here, however, mass spectrometry failed to identify specific methylation sites.

## 3. Discussion

**Figure 5:**
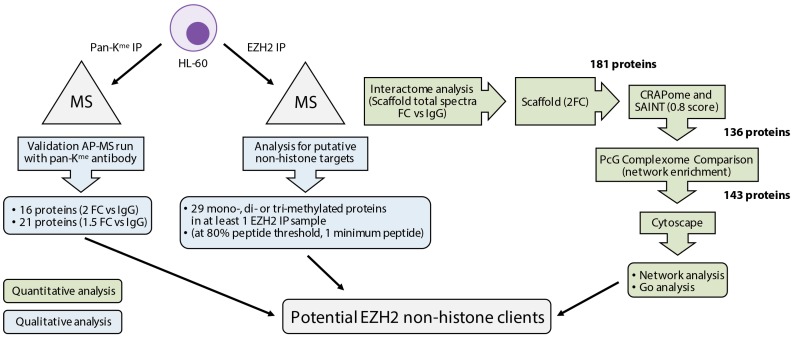
Summary of mass spectrometry (MS) strategy to identify potential non-histone clients in HL-60 cells.

In this study, we sought to enhance our understanding of the complex biological roles of the PRC2 complex in AML by analysing the EZH2 interactome (Figure 5). We found that previously established interactions with PRC2-recruiting proteins such as PHF1, PHF19, LCOR, JARID2, and EPOP are conserved in HL-60 cells and identified several proteins involved in transcriptional regulation including ALYREF, SFPQ, FLII, PARP1, and YBX1. We also found that EZH2 interacts with a number of RNA binding and processing proteins, including several that are implicated in stem cell maintenance such as YBX1 and DDX family proteins [52], suggesting a role for EZH2 in translational control. With regard to non-histone enzymatic substrates for EZH2, the identification of specifically methylated lysine residues and co-IP of the respective proteins with anti-EZH2 and anti-pan methyl lysine antibodies revealed several potential new targets. Importantly, many of the methylated lysine residues described in this study have been identified elsewhere [53,54], but without pointing to EZH2 as a candidate methyltransferase.

Although the exact biological role of methylation of ribosomal proteins remains poorly characterised, this post-translational modification is likely significant given that it is conserved in all three animal kingdoms [55]. It is well-established that both the 60S and the 40S subunits contain methylated residues and that a number of ribosomal proteins such as RPS2, 3, 9, 10, 12, 14, 25, and 27 as well as RPL23, 29, and 40 can also be methylated including on lysine residues [55]. Such modifications have been suggested to affect protein-protein interactions and ribosome assembly, RNA binding or translation accuracy [55]. In this context, the identification the eEF1A1 translation elongation factor as a substrate for EZH2 warrants further investigation. eEF1A1 lysine methylation has been described for several different residues, including K55 observed here [53,54]. Studies of EF-Tu (elongation factor thermo unstable), an *Escherichia coli* orthologue of eEF1A1, suggest that this conserved residue (K56 in *E. coli*) due to its location in the GTPase switch-1 region may enhance translational accuracy through attenuating GTP hydrolysis [55]. Given that PRC2 complexes have well-established functions in transcriptional regulation [56], our finding that EZH2 may be involved in translational control therefore expands the roles that PRC2 complexes play in the control of gene expression.

To date, therapeutic strategies targeting EZH2 have focused almost exclusively on inhibiting cofactor binding by the enzyme [18]. Even though effective when activating point mutations in the SET domain are driving lymphomas [57,58] or in the background of tumours harbouring other epigenetic perturbations [59], targeting the catalytic activity of EZH2 has thus far failed to elicit the expected response in a number of other malignancies [60,61]. Given that aberrant eEF1A1 activities have been implicated in a number of cancers [62,63,64] and the translation machinery represents an important area of oncology research [65], our findings suggest new possibilities for combinatorial therapeutic approaches.

## 4. Methods

### 4.1. Cell Culture and AtRA Treatment

HL-60 AML cells were cultured in RPMI-1640 (Gibco, Waltham, MA, USA) supplemented with penicillin (50 units/mL)/streptomycin (50 µg/mL) (Gibco, Waltham, MA, USA) and 10% fetal calf serum (Sigma-Aldrich, St. Louis, MO, USA). Cells were maintained at 37 °C and 5% CO_2_ in humidified atmosphere. AtRA was purchased from Sigma and was diluted in 1:1 ethanol and dimethyl sulfoxide (DMSO).

### 4.2. Affinity-Purification and Sample Preparation for Mass Spectrometry

HL-60 cells were centrifuged and washed with phosphate-buffered saline (PBS). Cell lysates prepared using High-salt Lysis Buffer (20 mM HEPES (4-(2-hydroxyethyl)piperazin-1-ylethanesulfonic acid) pH 7.9; 1.5 mM MgCl_2_; 0.3 mM NaCl; 0.2 mM EDTA (ethylenediaminetetraacetic acid); 0.1% Triton X100; 0.1 mM DTT (dithiothreitol); 25% Glycerol). Cell lysates were pre-cleared with protein A/G magnetic beads (Pierce) and incubated overnight at 4 °C with: rabbit polyclonal anti-EZH2 antibody (Abcam, ab186006, Cambridge, MA, USA); mouse monoclonal anti-EZH2 antibody (Cell Signaling Technology, clone AC22, Danvers, MA, USA); rabbit polyclonal anti-methyl lysine antibody (Abcam, ab7315); mouse IgG (Abcam, ab37355, Cambridge, MA, USA) or rabbit IgG (Abcam, ab27478, Cambridge, MA, USA) isotype controls. Samples were then incubated for 2 h with protein A/G magnetic beads. Bound antibody-protein complexes were washed three times with Low-salt Lysis Buffer (20 mM HEPES pH 7.9; 1.5 mM MgCl_2_; 0.1 mM NaCl; 0.2 mM EDTA; 0.1% Triton X100; 0.1 mM DTT, three times with 50 mM TEAB (triethylammonium bicarbonate), and eluted with 5% formic acid. Any residual formic acid was neutralised with 1 M TEAB and samples were dried *in vacuo*. Samples were re-dissolved in 5% acetonitrile/50 mM TEAB and then reduced with TCEP (tris(2-carboxyethyl)phosphine, 5 mM final concentration). Free cysteines were alkylated with 2-choloroacetamide (10 mM final concentration). Proteins were digested with trypsin (Promega, Madison, WI, USA) and quenched with neat formic acid after 4 h. An aliquot of these solutions was taken for direct analysis by liquid chromatography tandem-mass spectrometry (LC-MS/MS).

### 4.3. LC-MS/MS 

Reversed phase chromatography was performed using an HP1200 platform (Agilent, Santa Clara, CA, USA). Peptides were resolved on a 75 μm I.D. 15 cm C18 packed emitter column (3 μm particle size; Nikkyo Technos, Tokyo, Japan) over 30 min or 60 min using a linear gradient of 96:4 to 50:50 Buffer A:Buffer B [Buffer A (1% acetonitrile/3% dimethyl sulfoxide/0.1% formic acid), Buffer B (80% acetonitrile/3% dimethyl sulfoxide/0.1% formic acid)] at 250 nL/min. Peptides were ionised by electrospray ionisation using 1.8 kV applied immediately pre-column via a microtee built into the nanospray source. Samples were infused into an LTQ Velos Orbitrap mass spectrometer (Thermo Fisher Scientific, Waltham, MA, USA) directly from the end of the tapered tip silica column (6–8 μm exit bore). MS/MS data were acquired using data dependent acquisition based on a full Fourier Transform mass spectrometry (FT-MS) scan (30,000 resolution, inject time set to 500 milliseconds and Automatic gain control (AGC) set to 1,000,000 with preview mode disabled) and internal lock mass calibration against the ion 401.922718 *m*/*z*. The top 20 most intense precursor ions were fragmented by collision-induced dissociation and analysed using normal ion trap scans (AGC set to 30,000, normalised collision energy was set to 35% with an activation time of 10 milliseconds). Precursor ions with unknown or single charge states were excluded from selection. Peptides were measured in the orbitrap at 30,000 resolution (automatic gain control—AGC of 1,000,000). Peptides were then fragmented in the ion trap where they were measured at low resolution (AGC—30,000). Full FT-MS maximum inject time was 500 milliseconds and normalised collision energy was set to 35% with an activation time of 10 milliseconds. Wideband activation was used to co-fragment precursor ions undergoing neutral loss of up to −20 *m*/*z* from the parent ion, including loss of water/ammonia. MS/MS was acquired for selected precursor ions with a single repeat count acquired after 5-s delay followed by dynamic exclusion with a 10 ppm mass window for 10 s based on a maximal exclusion list of 500 entries.

### 4.4. Database Searching

Raw MS/MS data were submitted for database searching using Proteome Discoverer v1.4 and Mascot V2.3. The following Mascot search parameters were used: SwissProt Database, SwissProt_040511a (526,969 Sequences); taxonomy filter, Homo sapiens (20,305 sequences); enzyme specificity, trypsin (KR) 2 missed cleavages; mass tolerance; precursor 5 ppm, fragment 0.60 Da; variable modifications, acetyl (protein N-term); carbamidomethyl (C); oxidation of methionine pyro-Glu (peptide N-term Q); phosphorylation (STY); methylation (KR); dimethylation (KR); Trimethylation (K). MS/MS-based peptide and protein identifications were grouped and validated using Scaffold v4 (Proteome Software Inc. Portland, OR, USA). Protein identifications were automatically accepted if they contained at least two unique peptides assigned with at least 95% confidence by Peptide Prophet [66].

### 4.5. Data Analysis

All MS runs were merged in Scaffold 4.0 and assigned to two categories—IgG and EZH2 IP (or three categories for the initial analysis: IgG, EZH2 IP, and EZH2 IP in AtRA-treated cells). Fold change difference compared to IgG control (all IgG samples versus all EZH2 IP samples) were calculated using total spectra as a relative quantitation method (minimum value was set to 0.1 in case spectra were absent e.g., in IgG controls) and then exclusive spectrum count to eliminate potential spectra belonging to more than one protein (Table S1) [67]. Fisher’s exact test (with Benjamini-Hoechberg correction) was run in order to highlight potential hits. For the +/−AtRA analysis, a less stringent filter of 1.5-fold change over IgG was set and then the ratio of all four Untreated samples versus all three AtRA samples (two untreated versus two AtRA were matched biological samples) was compared. Proteins altering their relative abundance by a 2-fold change factor (either up or down) were also compared in the two matched +/−AtRA runs (in order to avoid false positive hits that pass the 2-fold change cut off, but have different direction in the two matched runs) and were finally checked to see if they were present in more than one sample. Hits from the global EZH2 interactome (all seven EZH2 IP samples versus all IgG controls) were further processed using the online analysis platform CRAPome [42]. The integrated Significance Analysis of INTeractome (SAINTexpress [43]) module was implemented to remove common contaminants (IgG selected as a criterion since the IP were of native non-overexpressed and non-tagged EZH2 protein) and to provide a relatively stringent confidence filter (score ≥ 0.8) for the probability of the bait-prey interactions. The resulting list of hits (136 proteins) was then enriched using a published PcG complexome MS data set [44] and the resulting data were processed, visualised, and analysed using Cytoscape (Cluster maker, MCODE, ClusterONE, and BinGO) as described previously [68]. USCF Chimera viewer [69] was used to visualise, process, and annotate the crystal structure of EZH2 (pdb: 5HYN) and eEF1A (pdb:4C0S).

## 5. Conclusions

In this study, we have generated the first EZH2 interactome in AML using an unbiased mass spectrometry approach. We identified EZH2 interactions with several ribosomal proteins, some of which are subject to change upon AtRA-induced myeloid differentiation. Our results strongly suggest that EZH2 is responsible for eEF1A1 K55 di-methylation, indicating a regulatory role in translation/mRNA processing that may present therapeutic opportunities.

## Figures and Tables

**Table 1:** STRING gene ontology.

GO Category	Description	FDR	Protein Name
GO.0006415	Translational termination	1.27 × 10^−43^	RPL10, RPL10A, RPL11, RPL12, RPL13, RPL13A, RPL15, RPL17, RPL18, RPL18A, RPL23, RPL24, RPL27A, RPL3, RPL35, RPL37A, RPL4, RPL5, RPL6, RPL7, RPL8, RPL9, RPLP0, RPLP1, RPLP2, RPS10, RPS12, RPS13, RPS15, RPS19, RPS2, RPS24, RPS27A, RPS3A, RPS4X, RPS6, RPS8
GO.0006414	Translational elongation	4.54 × 10^−42^	RPL10, RPL10A, RPL11, RPL12, RPL13, RPL13A, RPL15, RPL17, RPL18, RPL18A, RPL23, RPL24, RPL27A, RPL3, RPL35, RPL37A, RPL4, RPL5, RPL6, RPL7, RPL8, RPL9, RPLP0, RPLP1, RPLP2, RPS10, RPS12, RPS13, RPS15, RPS19, RPS2, RPS24, RPS27A, RPS3A, RPS4X, RPS6, RPS8
GO.0006413	Translational initiation	7.98 × 10^−41^	PABPC1, RPL10, RPL10A, RPL11, RPL12, RPL13, RPL13A, RPL15, RPL17, RPL18, RPL18A, RPL23, RPL24, RPL27A, RPL3, RPL35, RPL37A, RPL4, RPL5, RPL6, RPL7, RPL8, RPL9, RPLP0, RPLP1, RPLP2, RPS10, RPS12, RPS13, RPS15, RPS19, RPS2, RPS24, RPS27A, RPS3A, RPS4X, RPS6, RPS8
GO.0010467	Gene expression	2.09 × 10^−13^	AEBP2, AICDA, ALYREF, ANPEP, CBX3, DAPK3, DDX5, DMAP1, EED, EZH1, EZH2, FLII, FLNA, HNRNPF, HNRNPH1, HNRNPK, IGF2BP1, JARID2, LCOR, LRRFIP1, MTF2, NOLC1, NONO, PA2G4, PABPC1, PHF1, PHF19, RBBP4, RBBP7, RPL10, RPL12, RPL13, RPL17, RPL18, RPL18A, RPL24, RPL27A, RPL3, RPL35, RPL4, RPL5, RPL6, RPL7, RPL9, RPLP0, RPLP1, RPLP2, RPS10, RPS12, RPS13, RPS19, RPS2, RPS24, RPS27A, RPS3A, RPS6, RPS8, RRP1B, SFPQ, SND1, SRP14, SUZ12, U2AF2, YBX1
GO.0045892	Negative regulation of transcription	3.03 × 10^−2^	AEBP2, CBX3, DDX5, DMAP1, FLNA, HNRNPK, LCOR, LRRFIP1, MTF2, NONO, PA2G4, PARP1, RBBP7, RPS27A, SFPQ, SUZ12, YBX1

GO, gene ontology; FDR, false discovery rate.

**Table 2:** STRING gene ontology for AtRA-regulated EZH2 interacting proteins.

GO Category	Description	FDR	Protein Name
GO.0003723	RNA binding	4.86 × 10^−8^	ATP5A1, CCDC124, CORO1A, FLNA, HNRNPD, HNRNPF, ILF3, MSN, MTDH, PABPC1, PTBP1, RPSA, RRP1B

GO, gene ontology; FDR, false discovery rate.

**Table 3:** EZH2 interactome lysine methylation hits.

Lys monomethylation	**Protein Name**	**Peptide Sequence**	**Peptide Start Index**	**Peptide Stop Index**	**Variable Modifications Identified by Spectrum**	**Methylated Lys −AtRA**	**Methylated Lys +AtRA**
	CBX3	W**K**DSDEADLVLAK	142	154	K2: Methyl	12.5% (1/8)	ND (0/0)
	EZH2	YSQADAL**K**YVGIER	728	741	K8: Methyl	1.5% (2/131)	1.9% (2/106)
	Histone H1.2	**K**ASGPPVSELITK	34	46	K1: Methyl	2.1% (2/96)	5% (1/20)
	Histone H3.1	KSAPATGGV**K**PHR	28	41	K10: Methyl	0% (0/18)	16.7% (1/6)
	Histone H3.1	EIAQDF**K**TDLR	74	84	K7: Methyl	5.6% (1/18)	0% (0/6)
	MT1X	MDPNCSCSPVGSCAC-AGSCKC**K**ECKCTSCK	1	30	K22: Methyl	100% (1/1)	ND (0/0)
	RL36L	KQSGYGGQT**K**PIFR	44	57	K10: Methyl	33.3% (11/33)	44.4% (8/18)
	SUZ12	APQ**K**HGGGGGGGSGPSAGS-GGGGFGGSAAVAAATASGGK	2	40	K4: Methyl	1.3% (2/154)	0% (0/138)
Lys dimethylation	eEF1A1	GSF**K**YAWVLDK	52	62	K4: Dimethyl	11.9% (7/59)	5.9% (1/17)
	eEF1A1	MDSTEPPYSQ**K**R	155	166	K11: Dimethyl	0% (0/59)	5.9% (1/17)
	H3.1	**K**SAPATGGVKKPHR	28	41	K1: Dimethyl	5.6% (1/18)	50% (3/6)
	Histone H4	**K**VLRDNIQGITKPAIR	21	36	K1: Dimethyl	0% (0/86)	4.3% (1/23)
	MYO1D	S**K**DTCIVISGESGAGKTEASK	93	113	K2: Dimethyl	ND (0/0)	100% (3/3)
	RBP56	GPMTGSSGGDRGGF**K**	196	210	K15: Dimethyl	36.4% (8/22)	0% (0/6)
	TR150	DSRPSQAAGDNQGDEA**K**EQ-TFSGGTSQDTK	186	215	K17: Dimethyl	21.9% (7/32)	ND (0/0)
Lys trimethylation	ADT2	QY**K**GIIDCVVR	50	60	K3: Trimethyl	19.1% (4/21)	7.7% (1/13)
	HNRPQ	GGNVGG**K**R	558	565	K7: Trimethyl	25% (1/4)	16.7% (1/6)
	MT1X	MDPNCSCSPVGSCACAGSC-**K**CKEC**K**CTSC**K**	1	30	K20: Trimethyl, K25: Trimethyl, K30: Trimethyl	100% (1/1)	ND (0/0)
	ALYREF	AD**K**MDMSLDDIIK	2	14	K3: Trimethyl	0% (0/27)	5.2% (1/19)

**K**, methylated lysine; ND, not detected.

**Table 4:** Summary of potential non-histone EZH2 enzymatic targets.

Protein Name	Gene Symbol	Methylation Site	Protein Function
ADT2 (ADP/ATP Translocase 2)	*SLC25A5*	K52^me3^	ADP/ATP mitochondrial translocase
CBX3 (Chromobox 3)	*CBX3*	K142^me1^	Heterochromatin binding
eEF1A1 (Elongation factor 1-α1)	*EEF1A1*	K55^me2^, K165^me2^	Regulation of elongation
EZH2 (Enhancer of zeste homology 2)	*EZH2*	K735^me1^	Protein lysine methyltransferase
SUZ12 (Polycomb Repressive Complex 2 Subunit)	*SUZ12*	K4^me1^	Regulation of H3K27 methylation and gene expression
ALYREF (Aly/REF Export Factor)	*ALYREF*	K4^me3^	Chaperone of basic-region leucine zipper (bZIP) proteins

## Supplementary Materials

Supplementary materials can be found at www.mdpi.com/1422-0067/18/7/1440/s1.
