# Supplementary figures and images for: Semi-Quantitative Mass Spectrometry in AML Cells Identifies New Non-Genomic Targets of the EZH2 Methyltransferase
Source: Int J Mol Sci. 2017 Jul 5;18(7):1440. doi: 10.3390/ijms18071440 (PMC5535931; doi:10.3390/ijms18071440)

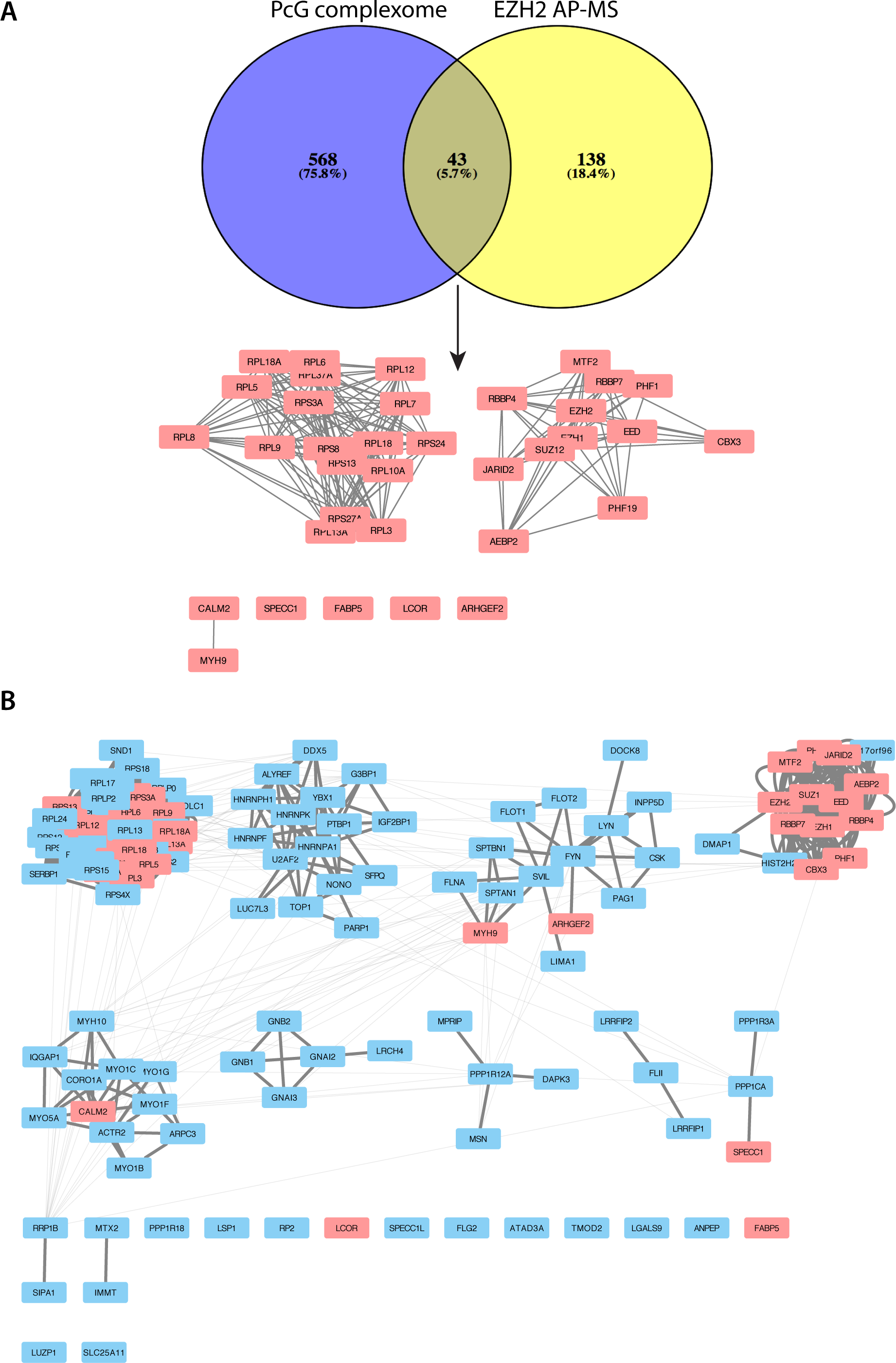

Supplement: Supplementary file 1 [file ijms-18-01440-s001.zip › ijms-205822-supplementary/Figure S1.tif]

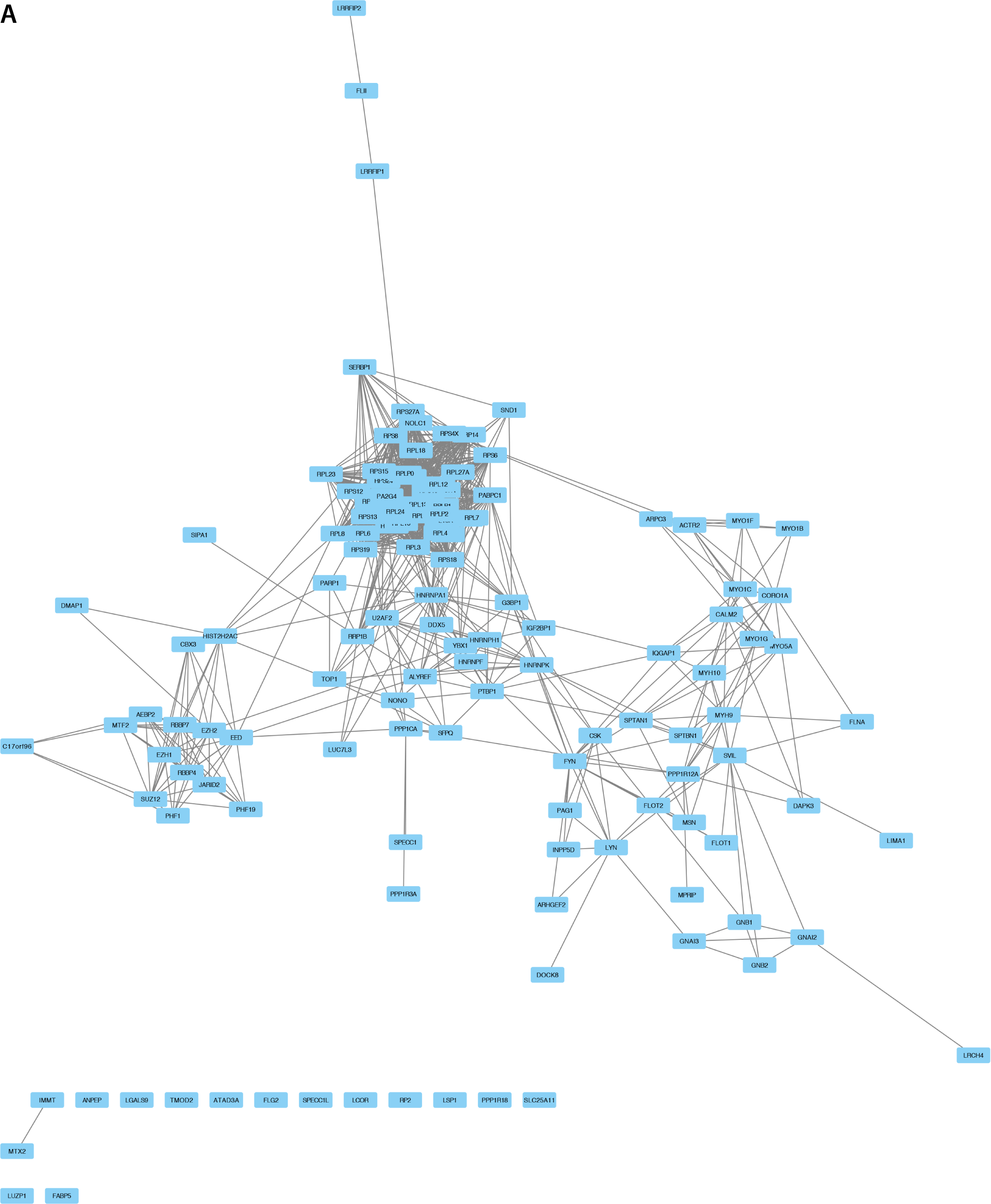

Supplement: Supplementary file 1 [file ijms-18-01440-s001.zip › ijms-205822-supplementary/Figure S2A.tif]

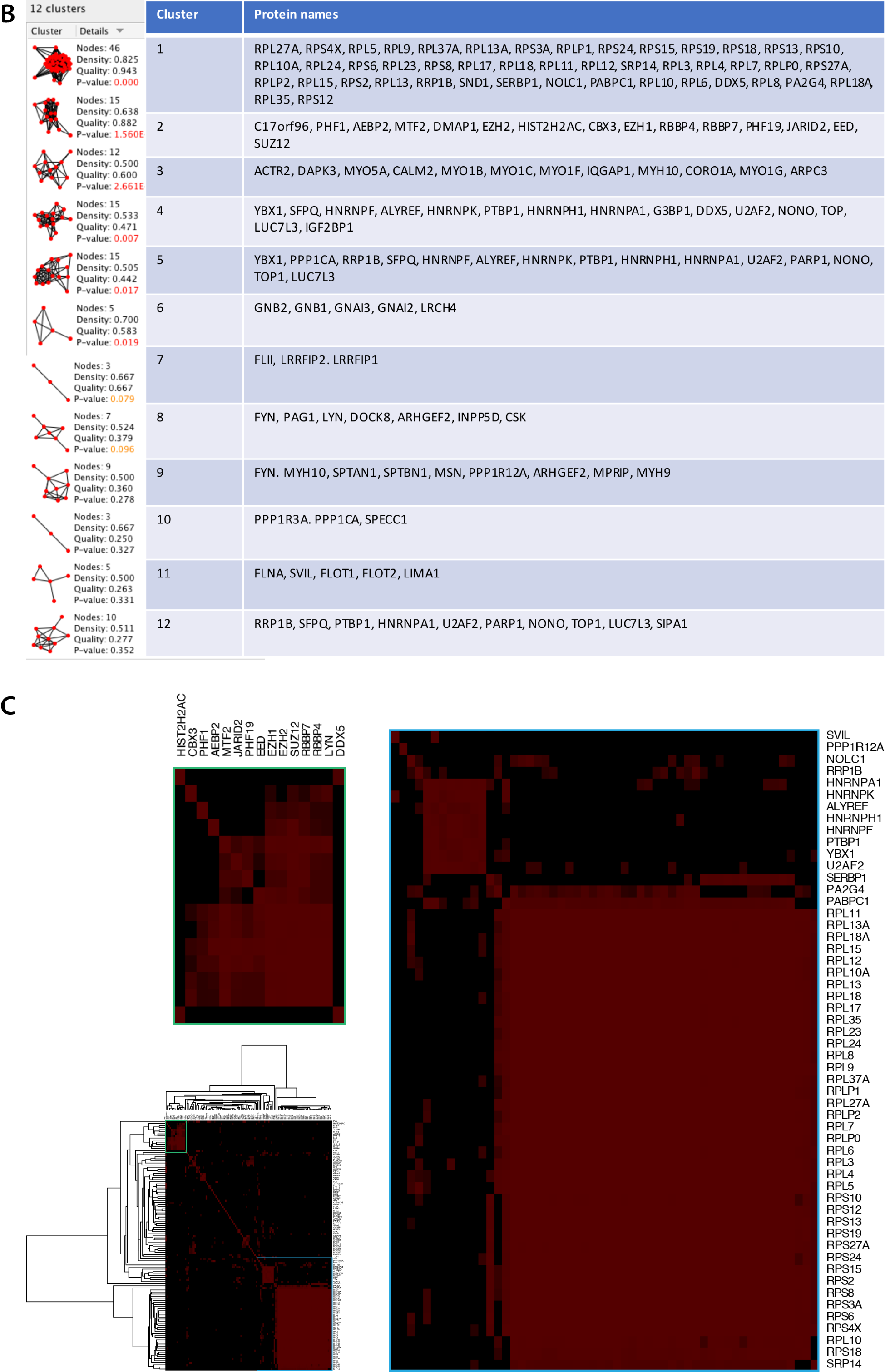

Supplement: Supplementary file 1 [file ijms-18-01440-s001.zip › ijms-205822-supplementary/Figure S2BC.tif]

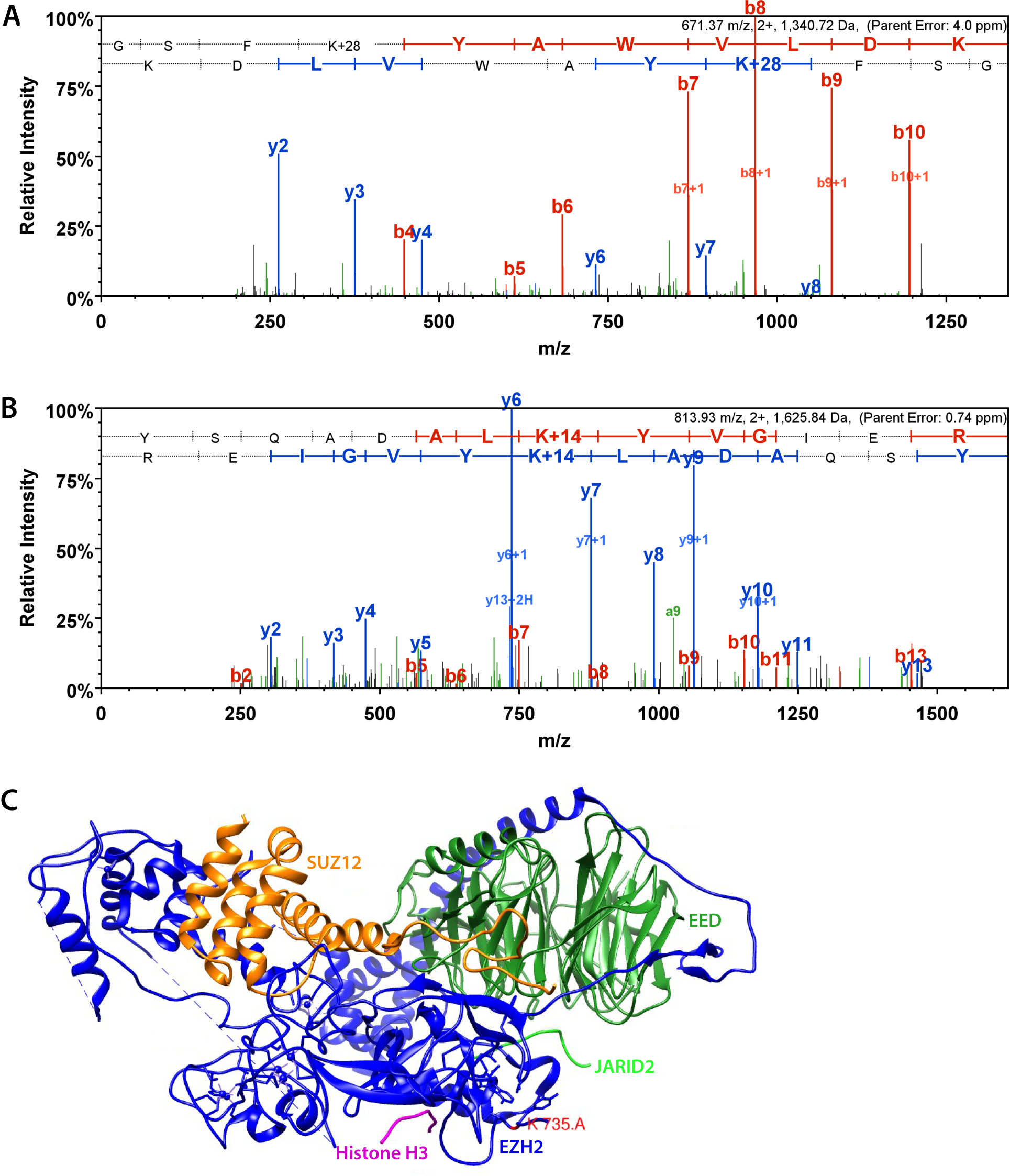

Supplement: Supplementary file 1 [file ijms-18-01440-s001.zip › ijms-205822-supplementary/Figure S3.tif]
